# Female patients with low systemic BMD

**DOI:** 10.3109/17453674.2010.537811

**Published:** 2010-11-26

**Authors:** WS Watson, K Periasamy, RMD Meek

**Affiliations:** Nuclear medicine department, Dept of Orthopaedics& Trauma, Southern General Hospital, Glasgow, U.K.

*Sir*—We read with interest the paper of Alm et al. (2009). The authors looked at post- total hip arhtroplasty periprosthetic (THA) bone changes in 39 subjects using DXA to analyse bone mineral density (BMD) in the Gruen zones surrounding the femoral prosthesis component. The purpose of the study was to look for factors such as bone mineral status that might influence femoral periprosthetic bone loss.

In Figure 4 of their paper, the authors graphed the change in BMD in Gruen zone 7 at 2 years post-THA expressed as a percentage of baseline BMD at that site (y-axis) against the lowest preoperative T score from lumbar spine, proximal femur or non-dominant distal forearm (x-axis). From the significant positive correlation they obtained between these graphed variables, they concluded that”low preoperative systemic BMD was associated with higher bone loss in Gruen zone 7”.

We would like to introduce a note of caution here because we will show that it is possible to demonstrate a significant correlation between the variables as defined by Alm et al when there is absolutely no influence of systemic BMD on postoperative BMD change at Gruen zone 7.

In order to do this we need to mimic their experimental data with “mock data” for both x and y variables as per Alm's Figure 4. From inspection of that figure, the x-axis T-scores appear to have a mean of around -1.5 and a standard deviation (SD) of 1. From this information, it is possible to produce a set of 39 random numbers normally distributed with this mean and SD using, for example, the “Data Analysis” toolkit in Microsoft Excel. This gives us our x variable. The y variable is comprised of a numerator showing the BMD difference between Gruen zone 7 BMD at 2 years and that at a preoperative baseline. From Alm et al's data in Table 2, there is an average Gruen zone 7 BMD loss of -0.25 g/cm^2^ over 2 years. We make the assumption that this is fixed at that value for all patients, i.e. no possibility of variation with systemic bone mineral status. The denominator of the y variable is the baseline Gruen zone 7 BMD expressed in g/cm^2^. From our own data on 26 adult females scheduled for THA, we obtain a regression equation for the excellent positive correlation of baseline Gruen zone 7 BMD against baseline Total Hip T-score as expressed below:


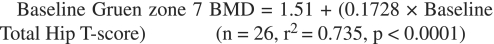


It is reasonable to accept this equation to an estimator for baseline Gruen zone 7 BMD given that Total Hip T-score is accepted as the most relevant indicator of systemic bone loss.

Therefore, we have constructed mock data for both the numerator and denominator of the y variable and can therefore combine these to provide “mock data” for the y variable. We now have a y variable incorporating a constant BMD change in Gruen zone 7. If we proceed to look for a “relation” between these “mock” x and y variables, we find that there is a strong positive correlation with the following regression equation:


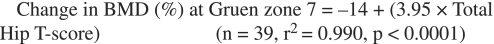


From inspection of Alm et al's Figure 4, the y-intercept is approximately -14 and the gradient is approximately 5.4. Therefore, our mock data gives similar results to the experimental data of Alm et al. This occurs despite our choosing a constant BMD change in Gruen zone 7 at 2 years postoperatively, i.e. a change that was independent of systemic bone mineral status. The reason we get a “false”positive correlation with our data is that the y variable denominator and the x variable, both of which are preoperative assessments, are highly correlated, i.e. a low Total Hip T-score corresponds to a low Gruen Zone 7 baseline BMD and because this appears on the denominator of the y variable, it increases the apparent % bone loss represented by that variable.

This analysis suggests that the conclusions made by Alm et al on the basis of their Figure 4 must be viewed with some caution. However, perhaps the most clinically important message from their study is that if you start with poor quality bone at the femoral prosthesis, any further postoperative loss whether related to systemic bone mineral status or not will not improve the situation.
