# Supplementary material for: Female patients with low systemic BMD
Source: Acta Orthop. 2010 Nov 26;81(6):768–9. doi: 10.3109/17453674.2010.537811 (PMC3216091; doi:10.3109/17453674.2010.537811)
Supplement: Related texts

# Letter: Female patients with low systemic BMD

**Published:** 2010-11-26

**Authors:** Jessica Alm, Hannu T Aro

**Affiliations:** Orthopaedic Research Unit, University of Turku, Turku, Finland, hannu.aro@utu.fi

*Sir*—We appreciate the concern posted by Drs. Watson, Periasamy and Meek considering the regression curve presented in our paper (Alm et al. 2009). We agree that it is important to be cautious when making conclusions from regression analyses because of the risk of false positive results. Here we present some points that verify our results and conclusions.

The main result and also the clinical message from our study was that systemic BMD predicts periprosthetic bone loss in Gruen zone 7, while other parameters (such as the local BMD of the hip) do not. This conclusion was made from the results presented in the line graph of Figure 3, where the patients were divided into three groups based on the systemic BMD (not the local BMD). We concluded from the results of this figure that “low preoperative systemic BMD was associated with higher bone loss in Gruen zone 7” since patients with systemic osteopenia or osteoporosis showed greater bone loss in this zone compared to patients with normal systemic BMD. We did not draw the conclusion based on the regression analysis presented in Figure 4. In fact, this figure was added as a request of a reviewer during the review process of the journal.

As posted by Watson and coauthors, it is possible to get a false positive correlation if the y variable denominator and the x variable are correlated. We would like to emphasize that the x variable used in Figure 4 was not the preoperative total hip T-score, but the lowest T-score recorded from all 10 areas measured for each patient, and in none of the 39 patients the lowest T-score was from the OA affected hip. Therefore, we did not have the problem caused by an expected high correlation between the total hip T-score and denominator of the y variable, i.e., the baseline BMD of Gruen zone 7.

What makes our results even more interesting is that when looking at the local BMD, i.e., the total hip T-scores, the values were high; only 6 of the osteopenic patients and one of the osteoporotic patients had osteopenic T-scores (between -1 and -2.5) at their affected hip. All other 32 patients had normal total hip T-scores. Still we found a significant association between the percentage change in BMD of Gruen zone 7 and the lowest systemic (not total hip) T-score. We can also add that the total hip T-scores did not correlate with the BMD-changes in Gruen zone 7 at 24 months.

Based on these results and those presented in our previous paper (Mäkinen et al. 2007), we disagree with Watson and Periasamy regarding local total hip T-score as the most representative for systemic bone loss. On the contrary, one of our main observations was that DXA measurement of the local BMD of the affected hip is not representative for systemic bone loss or a predictor for periprosthetic bone loss. Osteoarthritis of the hip joint seems to increase “artificially” the local BMD. A recent study (Lingard et al. 2010) also confirmed that the diagnosis of osteoporosis may be missed in patients awaiting total joint replacement unless BMD measurements are performed at sites distant from the joint affected by OA (such as non-dominant forearm).
